# Preferred Reporting Items for Overviews of Reviews (PRIOR): a protocol for development of a reporting guideline for overviews of reviews of healthcare interventions

**DOI:** 10.1186/s13643-019-1252-9

**Published:** 2019-12-23

**Authors:** Michelle Pollock, Ricardo M. Fernandes, Dawid Pieper, Andrea C. Tricco, Michelle Gates, Allison Gates, Lisa Hartling

**Affiliations:** 1 Institute of Health Economics, 1200 10405 Jasper Avenue, Edmonton, Alberta T5J 3N4 Canada; 2 Clinical Pharmacology Unit, Instituto de Medicina Molecular, Faculty of Medicine, University of Lisbon, Av. Professor Egas Moniz, 1649-028 Lisbon, Portugal; 3 Department of Pediatrics, Santa Maria Hospital, Lisbon, Portugal; 4 Institut für Forschung in der Operativen Medizin, Department für Humanmedizin, Universität Witten/Herdecke, Witten, Germany; 5 Li Ka Shing Knowledge Institute of St. Michael’s Hospital, 209 Victoria Street, Toronto, Ontario M5B 1T8 Canada; 6 Dalla Lana School of Public Health & Institute of Health Policy, Management, and Evaluation, University of Toronto, Toronto, Ontario Canada; 7 Queen’s Collaboration for Health Care Quality Joanna Briggs Institute Centre of Excellence, Queen’s University, Kingston, Ontario Canada; 8 Alberta Research Centre for Health Evidence, Department of Pediatrics, Edmonton Clinic Health Academy 4-482C, University of Alberta, 11405-87 Avenue NW, Edmonton, Alberta T6G 1C9 Canada

**Keywords:** Overviews of reviews, Healthcare interventions, Methods, Reporting, Reporting guideline

## Abstract

**Background:**

Overviews of reviews (i.e., overviews) compile information from multiple systematic reviews to provide a single synthesis of relevant evidence for healthcare decision-making. Despite their increasing popularity, there are currently no systematically developed reporting guidelines for overviews. This is problematic because the reporting of published overviews varies considerably and is often substandard. Our objective is to use explicit, systematic, and transparent methods to develop an evidence-based and agreement-based reporting guideline for overviews of reviews of healthcare interventions (PRIOR, Preferred Reporting Items for Overviews of Reviews).

**Methods:**

We will develop the PRIOR reporting guideline in four stages, using established methods for developing reporting guidelines in health research. First, we will establish an international and multidisciplinary expert advisory board that will oversee the conduct of the project and provide methodological support. Second, we will use the results of comprehensive literature reviews to develop a list of prospective checklist items for the reporting guideline. Third, we will use a modified Delphi exercise to achieve a high level of expert agreement on the list of items to be included in the PRIOR reporting guideline. We will identify and recruit a group of up to 100 international experts who will provide input into the guideline in three Delphi rounds: the first two rounds will occur via online survey, and the third round will occur during a smaller (8 to 10 participants) in-person meeting that will use a nominal group technique. Fourth, we will produce and publish the PRIOR reporting guideline.

**Discussion:**

A systematically developed reporting guideline for overviews could help to improve the accuracy, completeness, and transparency of overviews. This, in turn, could help maximize the value and impact of overviews by allowing more efficient interpretation and use of their research findings.

## Background

Overviews of reviews specific to healthcare interventions (sometimes referred to as “overviews of systematic reviews,” “reviews of reviews,” “reviews of systematic reviews,” or “umbrella reviews”; hereafter referred to as “overviews”) use explicit and systematic methods to search for, identify, extract data from, and analyze the results of multiple related systematic reviews (Table 1). Their aim is to provide a single synthesis of systematic review evidence to answer different types of questions related to the efficacy, effectiveness, and/or safety of healthcare interventions for preventing or treating various clinical conditions [1]. Because overviews have been gaining momentum as an increasingly popular knowledge synthesis product, methods for conducting overviews have evolved in recent years [2–6]. For example, in 2016, we published a scoping review summarizing existing guidance for conducting overviews of healthcare interventions [7], and we used the results to update the chapter on overview methods in the *Cochrane Handbook for Systematic Reviews of Interventions* [1]*.* Despite advances in methods for conducting overviews, the reporting of overviews varies considerably and is often substandard (Table 2) [2–4].

**Table 1:** Types of questions about healthcare interventions that overviews can examine^a^

1	Different interventions for the same condition or population.
2	Different approaches to the application of the same intervention for the same condition or population.
3	Same intervention for different conditions or populations.
4	Adverse effects of an intervention for one or more conditions or populations.
5	The same intervention for the same condition or population, where different outcomes or time points are addressed.

^a^From Pollock et al. 2019 Cochrane Handbook chapter on overviews of reviews [draft] [1]

**Table 2:** Percentage of overviews (published up to 2016 or 2017) reporting on key aspects of methods and results

Reporting item	Percentage of overviews reporting the item (%)
Indicate that they are working from a protocol [4]	22
Rationale [4]	60
Explicit statement of objectives [4]	56
PICO criteria for eligibility [4]	44
Primary outcome [28]	29
Databases and search dates [28]	74
Full search strategy [4]	36
Circumstances in which primary studies would be considered [4]	6
Description of methods used for all steps of screening [28]	69
Description of methods used for data extraction [28]	67
Description of methods used to assess quality or risk of bias [28]	78
Description of methods for addressing overlapping SRs in overviews [4]	44
Description of methods for addressing discordant SRs [4, 28]	4–6
Description of the synthesis methods	26
Description of included SRs (adequate detail to be replicable) [4]	20
Methodological quality of included SRs [4]	76
Methodological quality of primary studies contained within included SRs [4]	22
Certainty of evidence of outcome data [4]	34
Conflicts of interest statement [28]	82
Source of funding [28]	74

Data from Lunny et al. (*n* = 50 overviews) [4] and an ongoing review by Pieper et al. (*n* = 100 overviews) [28]

*SR* systematic review

A reporting guideline is defined as “a checklist, flow diagram, or explicit text to guide authors in reporting a specific type of research, developed using explicit methodology” [8]. To date, we are not aware of any published guidance that was developed based on the evidence-based and agreement-based process recommended by the Enhancing QUAlity and Transparency Of health Research (EQUATOR) Network [8]. We are, however, aware of several relevant documents that narratively describe (based on personal experience and principles of “good practice”) issues related to reporting overviews. Onishi and Furukawa [9] developed a checklist for overviews of reviews focused on clinical topics based on the recommendations of Cochrane [10], the Preferred Reporting Items for Systematic reviews and Meta-Analyses (PRISMA) statement [11], and the AMSTAR (A MeaSurement Tool to Assess systematic Reviews) quality assessment tool [12]. Bougioukas et al. [13] published a checklist (PRIO, Preferred Reporting Items for Overviews of Systematic Reviews) with specific emphasis on harms reporting that was designed by combining recommendations from PRISMA [11], PRISMA-harms [14], PRISMA-protocols (PRISMA-P) [15], and other relevant documents. The same research group published the first available guidance on the reporting of abstracts for overviews of reviews of healthcare interventions [16], generated by combining features of PRISMA for abstracts [17], the PRIO checklist [13], important features of published abstracts identified in their literature search, and expert input. Li et al. [18] produced a checklist for overviews of reviews of healthcare interventions based on the recommendations in the Cochrane handbook [10], PRISMA statement [11], and the Overview Quality Assessment Questionnaire (OQAQ) [19]. Finally, Singh [20] published a reporting checklist for metareviews (MARQ, Metareview Assessment of Reporting Quality checklist), a type of overview that descriptively compares the reporting characteristics of systematic reviews, based on AMSTAR [12] and Cochrane guidance [10].

An abstract for STROVI (Standards for Reporting of Overviews of Reviews and Umbrella Reviews statement) was presented at the 2017 Cochrane Colloquium [21], but our informal contact with the authors indicated that to date further work on the project has not been undertaken. We have included the primary author of the STROVI abstract, as well as authors of the other previously mentioned published checklists, as participants in our Delphi process, based on their interest and expertise in overview methodology. While the aforementioned guidance can be a good starting point for authors preparing overviews, there is a lack of clarity about which might be the best to use and under what circumstance. In addition, much of the evidence used to develop the guidelines has since been updated (e.g., a new Cochrane chapter on overviews was published [1], and AMSTAR 2 [22] was developed). There is a need for an up-to-date, rigorously developed [8] reporting guideline for overviews that can be endorsed by funders and publishers [8] to help authors report their methods and results in a consistent, complete, and transparent way [8, 23, 24]. This, in turn, could help end-users to better assess the reliability, validity, and applicability of overview results when making healthcare decisions [8, 25].

Our objective is to develop an evidence-based and agreement-based reporting guideline for overviews (PRIOR, Preferred Reporting Items for Overviews of Reviews) using explicit, systematic, and transparent methods, as recommended by the EQUATOR Network [8]. Because the unit of analysis differs from that of systematic reviews, we plan to develop the guideline de novo, rather than undertaking a PRISMA extension. Though we expect that some of the reporting guidance will be similar to PRISMA, the development of a stand-alone guideline will allow us to focus on the particular challenges in reporting overviews and to facilitate future guideline extensions (e.g., for diagnostic or prognostic accuracy overviews, etiology overviews). This guideline will consist of a “minimum essential set of items that should be reported” in overviews of healthcare interventions [8]. The PRIOR guideline will focus on overviews that examine the efficacy, effectiveness, and/or safety of healthcare interventions and that present narrative summaries and/or meta-analyses of quantitative outcome data. The target audience of the PRIOR reporting guideline will be overview authors, peer reviewers, journal editors, and healthcare decision-makers.

## Methods

**Fig. 1:**
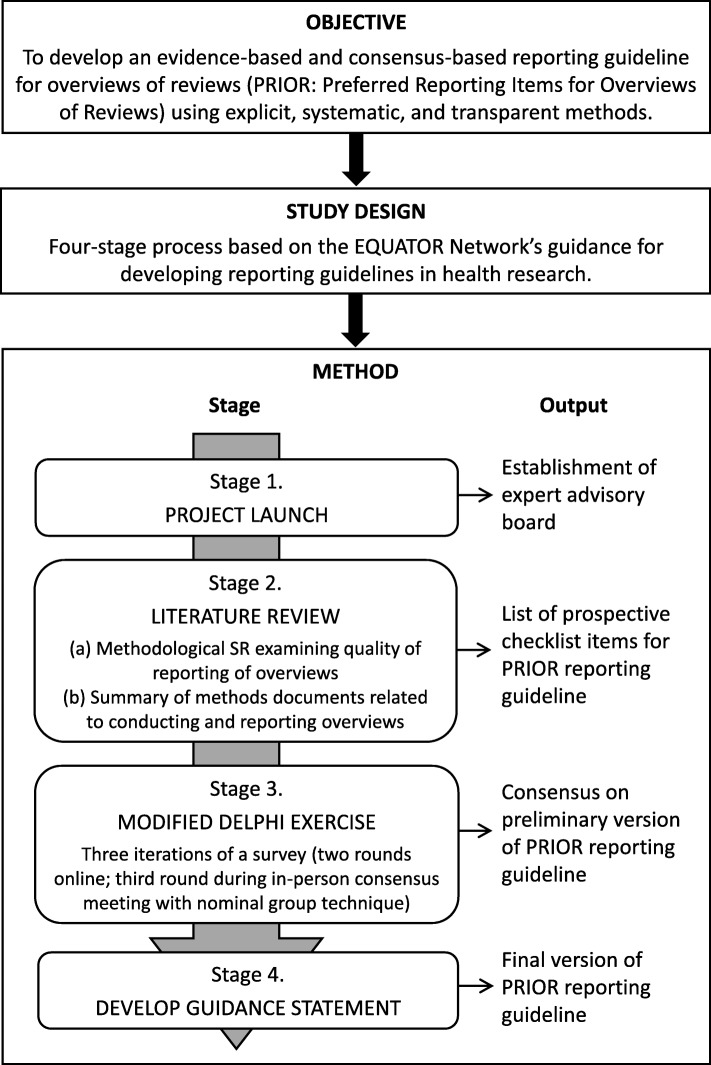
Four stages of the reporting guideline

This study will follow the key steps recommended by the EQUATOR Network for developing reporting guidelines in health research [8], which have been used to successfully develop reporting guidelines for other similar knowledge syntheses such as systematic reviews [11] and network meta-analyses [26]. We will develop the reporting guideline in four stages: (1) project launch, (2) literature review, (3) modified Delphi exercise, and (4) development of the guidance statement. These stages are illustrated in Fig. 1 and described below. We will record protocol amendments and describe these in our final manuscript. We have registered our intent to develop the PRIOR reporting guideline for overviews on the EQUATOR Network website (“reporting guidelines under development” section) [27]. The EQUATOR Network encourages registration of all reporting guidelines and extensions under development to raise awareness and help to prevent duplication. Before launching the project, we obtained ethics approval from the University of Alberta Health Research Ethics Board (#Pro00086094).

### Project launch

At project launch (December 2018), we established a core project team who will be responsible for the day-to-day operations of the planned project, consisting of methodologists with expertise in overview methodology and the development of reporting guidelines (MP [Canada]; LH [Canada]; RMF [Portugal]; DP [Germany]; ACT [Canada]), and research staff (AG and MG [Canada]).

We then established an international and multidisciplinary expert advisory board, with expertise in overview methodology and the development of reporting guidelines, who will advise on the development of PRIOR: Dr. David Moher (Canada); Dr. Tianjing Li (USA); Dr. Sue Brennan (Australia). The advisory board will be consulted regularly throughout the guideline development process. They will be asked to recommend relevant documents for the literature review; nominate participants for the Delphi exercise, review the checklist items for inclusion in the first round of the Delphi exercise, provide feedback after each round of the Delphi exercise (e.g., interpret results of the previous round, approve content for the next round), help plan and co-facilitate the in-person meeting, contribute to the production of the final reporting guideline, and assist with dissemination and knowledge translation activities.

### Literature review

To support the development of the PRIOR reporting guideline, we will conduct a methodological SR examining the quality of reporting of a sample of overviews of healthcare interventions published from 2012 to 2016 (in progress) [28]. To ensure that the most recently available data are used to inform the PRIOR guideline, we have supplemented our own data with those recently published by Lunny et al. in 2019 [4]. We will also search for and summarize methodological documents related to conducting and reporting overviews (e.g., documents that provide guidance for reporting overviews, documents that summarize methods used to conduct overviews, and empirical studies that evaluate methods for conducting overviews) (in progress). To identify relevant methodological documents, we will use and expand upon the most relevant search strategies contained within the scoping review by Pollock et al. [7], the methodological SR by Pieper et al. [28], and the evidence map by Lunny et al. [5, 6]. Our searches, which will continue until the final version of the PRIOR guideline is completed, will consist of: web searches (Google Scholar); reference tracking [29, 30]; monitoring article alerts; hand-searching websites, conference proceedings, and personal files; and asking experts (e.g., advisory board members; Delphi participants during the first survey round) for relevant articles. Two independent reviewers will assess titles and abstracts and all potentially relevant full-text articles for inclusion, with discrepancies resolved by consensus or third party adjudication. Results of the literature review will be used to develop and refine a list of checklist items that a panel of expert participants will assess for inclusion in the PRIOR reporting guideline.

### Modified Delphi exercise

A panel of experts (i.e., “participants”) will participate in a modified Delphi exercise to achieve a high level of agreement (≥ 70%) on the list of items to be included in the PRIOR reporting guideline [8]. The participants will provide feedback on potential items during three Delphi rounds, using structured feedback between rounds to help reconcile individual opinions and achieve group agreement [31–33]. Three rounds are likely to result in convergence of opinions between participants [32, 33]. The first two Delphi rounds will include all participants and occur online via self-administered survey; the third Delphi round will include a smaller subset of the participants (i.e., “expert panelists”) and occur during an in-person meeting that will use a nominal group technique (i.e., a formal consensus technique where expert panelists systematically review, discuss, and re-rate outstanding items) to achieve agreement [33, 34]. Should a high level of agreement (≥ 70%) not be reached following the in-person meeting, we may implement a third online survey among the expert panelists to assist in achieving agreement. Before the study, we will pilot test the survey’s usability, clarity, and face validity by sending it to five individuals familiar with overview methods but uninvolved in the current project. Their feedback will be used to revise the survey format and checklist items as needed.

### Participant recruitment

We will use a purposive sampling technique [31] to identify and recruit a panel of up to 100 participants with experience coordinating, conducting, reviewing, disseminating, and/or using overviews of healthcare interventions (e.g., editors, authors, peer reviewers, and end-users of published overviews such as guideline developers). We will aim to recruit international participants who have diverse roles (e.g., researchers, healthcare professionals, patients, journal editors, policy-makers, funding agency representatives) and are employed in a range of settings (e.g., universities, hospitals, government, non-profit organizations, for-profit organizations). Participants will form a single panel for the analysis of survey results, feedback between rounds, and criteria for agreement. A list of potential participants will be prioritized by the core project team, in order to provide representation from our populations of interest (i.e., editors, authors, peer-reviewers, end-users) and major evidence synthesis centers (i.e., Joanna Briggs Institute, Canadian Agency for Drugs and Technologies in Health, Campbell Collaboration, Cochrane, Agency for Healthcare Research and Quality Effective Health Care Program, Canadian Task Force on Preventive Health Care, Centre for Evidence-Based Medicine, Centre for Evidence-based Health Care). The core project team and expert advisory board will also provide input, based on their knowledge of the potential participants, on each participant’s level of expertise and potential for unique contribution to the development of the guideline (e.g., statistical expertise, information specialists). Participants will be invited via personalized email that will describe the PRIOR guideline development project and explain the objective, process, and timelines of the Delphi exercise. We will obtain informed consent from participants using an online consent form.

### Round one: Online survey

The participants will be asked to use a self-administered online survey to rate, on a four-point Likert scale, the extent to which they agree with the inclusion of each checklist item in the PRIOR reporting guideline (1 = strongly disagree, 2 = somewhat disagree, 3 = somewhat agree, 4 = strongly agree) [34]. Participants may also choose to answer “I don’t know” and provide an explanation [35]. For each item, a free text box will be provided for general comments (e.g., justification for their decision, proposed wording changes). Items will be presented in an order that reflects the progression of reporting in overviews (e.g., title, abstract, background, methods, results, discussion, other). At the end of the survey, two free text boxes will be provided for participants to suggest additional checklist items, and relevant methods papers. The Dillman principles for constructing respondent-friendly web surveys will be used to design the survey and its component items [36, 37]. Round one of the survey will remain open for 1 month (February–March 2020), during which bi-weekly reminder emails will be sent. The survey will be completed quasi-anonymously (i.e., the core project team but not the other study participants will know the identities and responses of the participants [31]), using a versatile online Delphi platform (e.g., Welphi). We will collate and summarize survey results, with agreement defined a priori as ≥ 70% for inclusion in (i.e., score of 3–4) or exclusion from (i.e., score of 1–2) the reporting guideline [38], based on the total number of responses obtained per question.

### Round two: Online survey

In the second online survey (June–July 2020), the same participants will view and/or re-rate the checklist items presented in the first survey [32, 33]. The content, structure, and process will be similar to that of the first survey, with two differences. First, checklist items may be re-worded and/or re-formatted (e.g., items may be split or combined) based on the free-text comments collected in the first survey. Second, we will provide participants with their individual responses and a summary of anonymized group ratings, including all free-text comments, from the first survey. Participants will be asked to consider the structured feedback to inform their responses [32, 33]. Checklist items will be presented in the same order as previously. The items that reached agreement (≥ 70%) after the first survey will be presented for information purposes only (i.e. no more voting will occur, though participants may respond to free-text comments). The items that did not reach a high level of agreement during the first survey will be re-rated. The participants will be asked to determine whether and how they wish to modify their original answers in light of the group responses and comments; they may also provide feedback on free-text comments if desired. At the end of the survey, we will ask the participants to rate and provide comments for each new checklist item generated by participants during the first survey. We will collate and summarize survey results, with agreement defined as previously described (≥ 70%).

### Round three: In-person meeting

With input from the advisory board, we will select and invite a minimum of 8 to 10 participants (i.e., “expert panelists”), ideally representing different stakeholder groups, to convene at a 1-day, in-person meeting (October 2020), where a nominal group technique and real-time voting will be used to systematically discuss and resolve outstanding disagreements [8, 33, 34]. If needed, a subset of these expert panelists may participate remotely using interactive software that allows for real-time screen sharing, audio discussion, and user comments (e.g., Adobe Connect, GoToMeeting). Moderators with expertise in overview methods and previous experience conducting consensus meetings for reporting guidelines will facilitate the meeting. We will audio record the meeting and take meeting minutes. The objective of the meeting will be to obtain final agreement on the list of items to be included in the PRIOR reporting guideline.

Prior to the meeting, members of the expert panel will receive a copy of their second-round survey results. We will begin the meeting by briefly summarizing the items reaching agreement for inclusion in, and exclusion from, the reporting guideline. No further voting will occur for these items, but outstanding free-text comments will be presented and resolved as needed; this could include rewording or reformatting of existing items. The bulk of the meeting will use a nominal group technique to obtain a high level of agreement (i.e., ≥ 70% agreement, as previously described) on those checklist items still lacking agreement [33, 34]. Each item will be reviewed sequentially, using a three-step process. First, facilitators will present the group survey results, all free-text comments, and all relevant methodological literature related to each item. This can help structure the interaction, provide a common starting point for participants, and promote evidence-based discussions about guideline content [33]. Second, the expert panel will discuss, debate, and aim to resolve discrepancies in a structured large-group discussion [33, 34]. Third, expert panelists will vote on the inclusion or exclusion of remaining items from the guideline.

The expert panel will be asked to re-rate the extent to which they agree with the inclusion of each checklist item. The content, structure, and process of the survey will be similar to the online surveys, with two changes: (1) the “I don’t know” option will no longer be available and (2) no free-text comments will be solicited. Expert panelists will complete the survey anonymously using a secure, online, live voting platform (e.g., Sli.do) that they will access using their personal electronic devices, with agreement defined as previously described (≥ 70%). Aggregate survey results will be automatically compiled by the software and presented to the group at the end of the survey. If agreement has not been reached on all items following the survey, we will ask the expert panelists to divide themselves into one small group per checklist item. Each small group will engage in unmoderated discussion to achieve final agreement, with rationale, for each outstanding item. Post-meeting discussions may continue over email or teleconference (i.e., including the expert panelists and facilitated by the core team) to achieve agreement, or we may implement an additional online survey with expert panelists, if needed. The meeting will conclude by discussing the strategy for producing, publishing, and disseminating the final guideline. We will discuss the inclusion of a flow diagram, development of an accompanying explanation and elaboration document, who will be involved in which activities, and publication and knowledge translation strategies [8].

### Development of the guidance statement

A small writing group will iteratively draft the final version of the PRIOR guidance document based on the final decisions of the expert panel. The writing group will consist of the core project team, with an open invitation issued to the advisory board members and expert panel members. We will aim to provide clear, concise, and unambiguous wording for each PRIOR checklist item. The reporting guideline will be circulated among all advisory board members and expert panel members to obtain final input and approval prior to publication.

## Discussion

Despite the growing number of published overviews and the commonly observed deficiencies in reporting of overviews [2–4], there are currently no systematically developed reporting guidelines for overviews of healthcare interventions. This protocol will help address this gap in guidance by using a four-stage process to develop the PRIOR reporting guideline, an evidence-based and agreement-based reporting guideline for overviews of healthcare interventions. Once completed, we will submit the PRIOR reporting guideline for publication to an appropriate peer-reviewed journal (we may also seek co-publication in multiple journals, if appropriate). The guideline will also be published on the EQUATOR website and the Cochrane Comparing Multiple Interventions Methods Group (CMIMG) website. We will aim to present the guideline at conferences and workshops, disseminate the guideline via email lists, and solicit journal editors to actively endorse the guideline. Other potential methods that we will use to disseminate the guideline could include videos, infographics, and social media.

The PRIOR reporting guideline will be developed using a modified Delphi process, which is commonly used to develop reporting guidelines in the health sciences [11, 26]. There are established benefits to using a modified Delphi process. For example, the online surveys provide a time- and cost-effective way to obtain preliminary agreement, while the more intensive in-person meeting with nominal group technique allows for face-to-face, in-depth discussion of outstanding issues [33]. Using an explicit, controlled, and scientifically credible process to achieve a high level of agreement among a group of experts can help to leverage the benefits of individual expertise and group decision-making, while simultaneously minimizing the biases associated with informal decision-making [33]. This can enhance the credibility of the guideline development process and help to ensure widespread acceptance and uptake of the reporting guideline. We expect some participant attrition between Delphi rounds; we will minimize this by following Dillman’s principles [36, 37] and conducting pilot tests to enhance the usability of the survey, providing bi-weekly email reminders, and choosing a survey platform that supports participant convenience by allowing them to leave and return at any time. Additionally, the PRIOR reporting guideline will only capture current expertise based on the existing state of knowledge, and we expect that requirements for reporting overviews will evolve over time as overview methods evolve. Thus, the aim of the PRIOR reporting guideline will not be to provide a definitive or unchanging list of reporting requirements, but rather to capture current expertise and knowledge upon which future research can build.

Once completed, the planned work will result in an evidence-informed, consensus-based reporting guideline for overviews of reviews of healthcare interventions. The PRIOR reporting guideline will help overview authors improve the accuracy, completeness, and transparency of reporting. It will also provide a framework for peer reviewers, journal editors, and healthcare decision-makers to critically appraise submitted or published overviews. Strengthening the reporting of overviews can help healthcare decision-makers better evaluate the reliability, validity, and applicability of overview results. This, in turn, can maximize the impact of overviews by allowing more accurate interpretation and use of their research findings.

## Data Availability

Not applicable.

## Abbreviations

PRIOR: Preferred Reporting Items for Overviews of Reviews
SR: Systematic review
